# Dimensions of poverty as risk factors for antimicrobial resistant organisms in Canada: a structured narrative review

**DOI:** 10.1186/s13756-022-01059-1

**Published:** 2022-01-24

**Authors:** Teagan King, Richelle Schindler, Swati Chavda, John Conly

**Affiliations:** 1 Department of Medicine, Cumming School of Medicine, University of Calgary and Alberta Health Services, Calgary, AB Canada; 2 Medical Officer of Health, Alberta Health Services, Calgary, AB Canada; 3 Provincial Population and Public Health, Alberta Health Services, Calgary, AB Canada; 4 Department of Microbiology, Immunology and Infectious Diseases, Cumming School of Medicine, University of Calgary, Calgary, AB Canada; 5 Department of Pathology & Laboratory Medicine, Cumming School of Medicine, University of Calgary and Alberta Health Services, Calgary, AB Canada; 6 O’Brien Institute for Public Health, University of Calgary and Alberta Health Services, Calgary, AB Canada; 7 Synder Institute for Chronic Diseases, University of Calgary and Alberta Health Services, Calgary, AB Canada

**Keywords:** Antimicrobial resistant organisms, Poverty, Income, Canada, Risk factors, Narrative review

## Abstract

**Background:**

Few studies have assessed the relationship between poverty and the risk of infection with antimicrobial resistant organisms (AROs). We sought to identify, appraise, and synthesize the available published Canadian literature that analyzes living in poverty and risk of AROs.

**Methods:**

A structured narrative review methodology was used, including a systematic search of three databases: MedLINE, EMBASE and Web of Science for articles pertaining to poverty, and infection with AROs in Canada between 1990 and 2020. Poverty was broadly defined to include economic measures and associated social determinants of health. Based on inclusion and exclusion criteria, there were 889 initial articles, and 43 included in the final review. The final articles were extracted using a standard format and appraised using the Joanna Briggs Institute Levels of Evidence framework.

**Results:**

Of 43 studies, 15 (35%) related to methicillin-resistant *Staphylococcus aureus* (MRSA). One study found a 73% risk reduction (RR 0.27, 95%CI 0.19–0.39, p =  < 0.0001) in community-acquired MRSA (CA-MRSA) infection for each $100,000 income increase. Results pertaining to homelessness and MRSA suggested transmission was related to patterns of frequent drug use, skin-to-skin contact and sexual contact more than shelter contact. Indigenous persons have high rates of CA-MRSA, with more rooms in the house being a significant protective factor (OR 0.86, *p* = 0.023). One study found household income over $60,000 (OR 0.83, *p* = 0.039) in univariate analysis and higher maternal education (OR 0.76, 95%CI 0.63–0.92, *p* = 0.005) in multivariate analysis were protective for otitis media due to an ARO among children. Twenty of 43 (46.5%) articles pertained to tuberculosis (TB). Foreign-born persons were four times more likely to have resistant TB compared to Canadian-born persons. None of the 20 studies used income in their analyses.

**Conclusions:**

There is an association between higher income and protection from CA-MRSA. Mixed results exist regarding the impact of homelessness and MRSA, demonstrating a nuanced relationship with behavioural risk factors. Higher income and maternal education were associated with reduced ARO-associated acute otitis media in children in one study. We do not have a robust understanding of the social measures of marginalization related to being foreign-born that contribute to higher rates of resistant TB infection.

**Supplementary Information:**

The online version contains supplementary material available at 10.1186/s13756-022-01059-1.

## Background

Antimicrobial resistance is a growing area of importance in clinical and public health research. In Canada in 2018, there were an estimated 250,000 antimicrobial resistant infections, resulting in 14,000 deaths [1]. The issue of rising resistance has far-reaching impacts on individuals in terms of morbidity and mortality, health care delivery, and the Canadian economy more broadly. As is the case for many health issues, vulnerable groups tend to experience a disproportionate burden of disease. We aimed to better understand health inequities related to poverty and risk of infections with antimicrobial resistant organisms (AROs).

Our current understanding of poverty as a risk factor for infection or colonization with resistant organisms in Canada is limited. Internationally, a systematic review conducted by Alividza and colleagues in 2018 [2] outlines a complex relationship between the dimensions of poverty, including income, housing quality, water quality and education level, with rates of AROs. In high-income countries, their results suggested a positive association between poor housing, lack of education and low income and infections with methicillin-resistant *Staphylococcus aureus* (MRSA), resistant *Streptococcus pneumoniae* and carbapenem-resistant organisms.

The 2019 Canadian Council of Academics report *When Antibiotics Fail*, which cites many supporting studies relevant to our objectives [1], outlines current recognized socio-demographic risk factors of resistant infections in Canada: crowded living conditions, homelessness, incarceration, occupation, birth country, and Indigenous status, among others. Many of these factors intersect with poverty and measures of socioeconomic deprivation. They show that Indigenous status has been associated with higher rates of community-acquired MRSA, but there has been no attempt to separate Indigeneity from the impacts of systemic and colonial violence on Indigenous communities in Canada. They also describe that while other infections, such as susceptible tuberculosis, pneumococcal disease, sexually transmitted infections, and gastrointestinal infections, have a higher prevalence among Indigenous peoples, there does not appear to be an elevated rate of resistance for these infections within these groups [1]. Further, the report notes that living conditions, homelessness and incarceration do not appear to be risk factors for resistant tuberculosis in Canada. Altogether, the current available literature has yet to provide a comprehensive understanding of the relationship between the dimensions of socioeconomic disadvantage and infections with AROs in Canada.

The objective of this narrative review was to identify, appraise and synthesize the available published Canadian literature examining the relationship between living in poverty, or experiencing a dimension of poverty as defined by Statistics Canada [3], with risk of infection or colonization with AROs. The overall aim was to contribute to the current understanding of the aspects of poverty that predispose vulnerable populations to infections with resistant organisms. Having a grasp of the current state of knowledge will allow us to better define the clinical and social dimensions of antimicrobial resistance (AMR) as we seek to address it.

## Methods

We conducted a structured narrative review to examine our question of interest, given it allowed us both to create a replicable, structured study with predefined inclusion and exclusion criteria, along with the ability to appraise a wide body of literature from which to draw conclusions (5). A detailed search strategy was developed in conjunction with a library scientist and is available in the supplemental material (see Additional file 1). Three databases were selected for the literature search: MedLINE, EMBASE and Web of Science. These databases encompass the dominant public health and biomedical literature, allow a complex search strategy, and include databases that are indexed (MedLINE, EMBASE) and a non-indexed, interdisciplinary database (Web of Science). We developed pre-defined inclusion and exclusion criteria to improve the replicability of our study. Articles that were considered in scope of this review met the following criteria: addressed the issue of antimicrobial resistant infections in humans; study population in Canada only; demonstrated link to a poverty indicator; and pertained to infection or colonization with any resistant bacterial organism, at any anatomical site, of any clinical significance. Articles needed to be in English or French, with full-text available, original research, and published between 1990 and 2020. We included adult and pediatric populations, with the latter being older than one year. We defined original research as any type of observational or experimental study, and excluded commentaries, opinion pieces, conference abstracts or any other incomplete review that did not specify a particular methodology. The definition of antimicrobial resistance was accepted if the authors provided a sound explanation of their methods. Articles that focused on environmental isolates, zoonotic, parasitic or protozoal infections, or antiviral, or antifungal resistance were excluded from this review.

The initial articles from the search underwent title and abstract screening by two independent reviewers (TK and SC), with conflicts resolved by the research team by area of expertise (JC and RS). The subsequent set of articles underwent full-text screening for final inclusion. Articles selected for final inclusion underwent a standardized data extraction process and quality assessment process, which is discussed in detail in the Quality Assessment section.

A review of the grey literature was also developed to review data from key governmental associations that report on AROs with Canadian demographic data. We used a keyword-based search with the following terms: antimicrobial resistance AND poverty; or antibiotic resistance AND poverty (résistance aux antibiotiques ET pauvreté). The search focused on the federal health organisations, including the Public Health Agency of Canada, and the respective provincial health authorities across Canada. Details of the grey literature search methodology can be found in Additional file 2.

### Quality assessment

The articles for inclusion were extracted using a standardized data form in *Microsoft Excel version 16.51,* which detailed the study participants, study design, study findings, poverty indicator and relationship to data regarding AROs. The level of evidence for each article was appraised using the Joanna-Briggs Institute (JBI) Levels of Evidence framework [4]. Recognizing the dimensions of poverty vary in the strength of their relationship to income, we developed a composite quality measure that included the JBI level of evidence and the strength of the poverty indicator (Fig. 2). Each article was then assigned a composite quality rating of low, medium or high. The rating is a measure of the overall ability of the study to answer our study question: i.e., was poverty, or a strong indicator of poverty, linked to a higher rate of infection with an ARO, considering the level of evidence of the article. For instance, a rating of high would suggest a high-quality study that used income in their analysis.

**Fig. 1:**
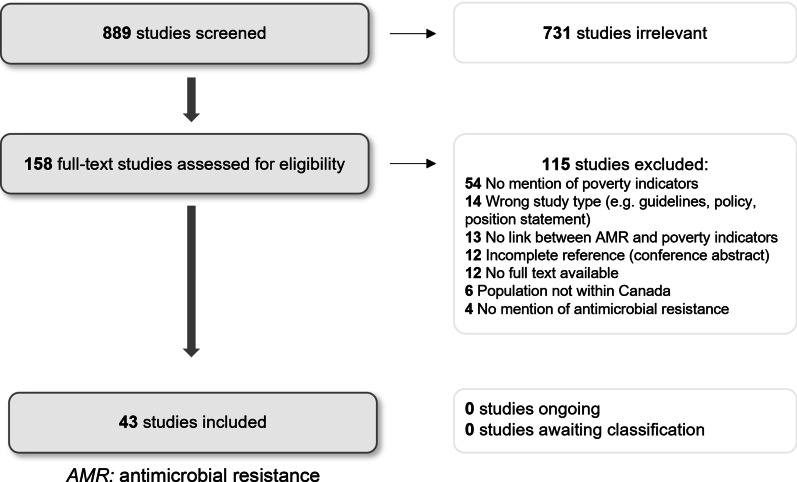
Study PRISMA diagram. *AMR:* antimicrobial resistance

**Fig. 2:**
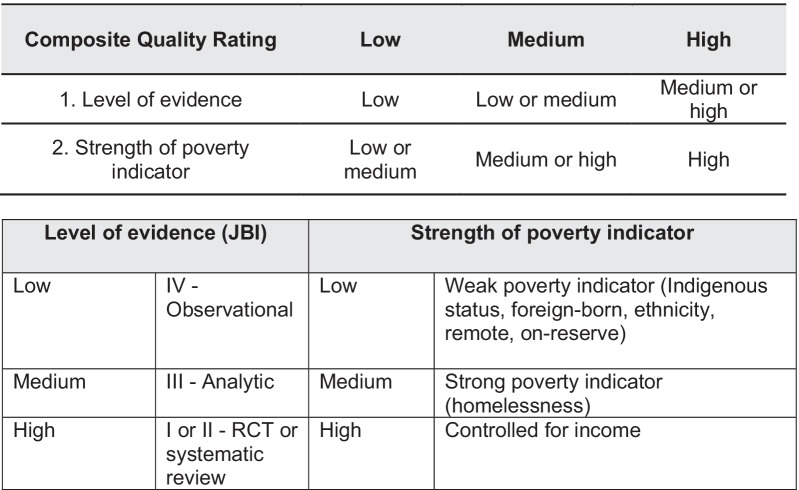
Composite quality rating (quality + strength of I\indicator)

### Poverty indicators

For the purposes of our review, we considered ‘poverty’ to be a broad condition of social and material deprivation. To gain a fulsome understanding of what is known about AROs and experiences of poverty in Canada, we adapted a complex definition of poverty using the Statistics Canada Dimensions of Poverty Hub framework [3]. This framework recognizes many indicators of poverty, or the socio-demographic risk factors that contribute to, or are a result of, social and material deprivation. The poverty indicators included in our definition of poverty were: low income, homelessness, contact with corrections or incarceration, crowded living, food insecurity, living on a reserve or living in a remote community, Indigenous status, being foreign-born or an immigrant or refugee, and low literacy or educational attainment.

While many of these chosen indicators are not synonymous with poverty, systemic barriers faced by marginalized groups in Canada leads to a disproportionate experience of poverty. This may be true for Indigenous groups, immigrants and refugees, and people who are unstably or under housed. Too often, marginalized identity status is the only poverty indicator examined in biomedical research, and as such, we have included these surrogate indicators of poverty in our analysis as reflections of the full spectrum of poverty in Canada.

## Results

The initial search yielded 889 articles. These articles were independently screened by two reviewers, leading to the selection of 158 papers for full-text review. Based on the full-text review against our inclusion and exclusion criteria, 43 articles were included in the final analysis (Fig. 1). Following extraction and quality appraisal, the included articles revealed one high quality article, 8 medium quality articles and 34 low quality articles based on our composite quality measure. The targeted grey literature review did not yield any additional literature for inclusion.

Of the 43 articles selected for inclusion in the final analysis, 15 (34.8%) articles pertained to MRSA, 20 (46.5%) to tuberculosis, five (11.6%) to invasive bacterial diseases, one to ARO-associated acute otitis media, one to *Helicobacter pylori (H. pylori),* and one to gonorrhea*.* The results are summarized by pathogen or disease state. Each section contains a summary table with the study findings, level of evidence, and composite quality measure, reported in Tables 1, 2, 3, 4, 5, 6.

**Table 1:** Studies exploring relationship between poverty indicators and MRSA

References	Location	Participants	Type of study	Infection/ colonization	Poverty indicator used	Association of resistance with poverty indicator	Quality level (JBI)	Composite rating
Daley [5]	NL	442	Cross-sectional	CA- & polyclonal MRSA colonization	Indigenous, correctional contact, homeless	Less crowded protective, homeless negative, correctional negative	IV	Med
Dalloo [6]	NWT	43	Outbreak investigation	CA-MRSA infection	Inuit, remote	High rate in community	IV	Low
Embil [7]	MB, SK, AB	259	Retrospective review	Polyclonal MRSA infection or colonization	Indigenous	Indigenous positive, rural positive	IV	Low
Gilbert [8]	AB	40	Retrospective review	CA-MRSA infection	Homeless, drug use, & correctional contact grouped	High-risk grouping positive	IV	Med
Gilbert [9]	AB	271	Cross-sectional	CA-MRSA colonization or infection	Homeless or correctional contact	Homeless negative, correctional negative, behaviors positive	IV	Med
Gill [10]	AB	4521	Retrospective review	CA-MRSA infection	Income	Income protective	IV	Med
Jeong [11]	AB, SK, MB, ON, QC	372	Retrospective review	CA-MRSA SSTIs	Indigenous, on-reserve	High rate on reserve	IV	Low
Kirlew [12]	ON	23	Case series	CA-MRSA bacteremia	Indigenous, on-reserve	High rate in community	IV	Low
Li [13]	AB	2581	Retrospective review	Lab isolates CA-MRSA	Indigenous, correctional facility	Indigenous positive, corrections negative	IV	Low
Main [14]	ON	10	Outbreak investigation	CA-MRSA infection	Correctional facility	Correctional contact positive	IV	Low
Muileboom [15]	ON	2451	Retrospective review	CA-MRSA infection	Indigenous, remote, rural	High rate in community	IV	Low
Ofner-Agostini [16]	Canada	148 Indigenous; 3589 Non-Indigenous	Retrospective review	Polyclonal MRSA infection or colonization	Indigenous	Indigenous positive	IV	Low
Szakacs [17]	ON	84	Cross-sectional	HA-MRSA colonization	Homeless	Homeless positive	IV	Med
Vayalumkal [18]	ON	153	Case–control	CA- & HA-MRSA infection	Homeless, correctional contact, Indigenous	Homeless positive, correctional positive	III	Med
Wylie [19]	MB	324	Retrospective review	HA- & CA-MRSA infection	Indigenous, on-reserve	No association	IV	Low

*AB:* Alberta; *CA:* Canada*; CA-MRSA:* Community-acquired Methicillin-Resistant *Staphylococcus aureus; HA-MRSA:* Hospital-acquired MRSA; *MB:* Manitoba; *SK:* Saskatchewan; *SSTI:* skin and soft tissue infection; *NL:* Newfoundland; *NWT:* Northwest Territories; *ON:* Ontario; *QC:* Quebec

**Table 2:** Studies exploring relationship between poverty indicators and tuberculosis

References	Location	Participants	Type of study	Type of infection	Poverty indicator used	Association of resistance with poverty indicator	Quality level (JBI)	Composite rating
Avendano [21]	Toronto, ON	40	Case series	MDR-TB	Foreign-born	Prior history of TB positive, higher prevalence foreign-born	IV	Low
CCDR [20]	Canada	1634	Cross-sectional	Any TB infection	Foreign-born	Foreign born increased TB prevalence, positive for resistant-TB	IV	Low
Cook [22]	MB	147	Cross-sectional	TB lymphadenitis	Foreign-born, Indigenous	Foreign born and Indigenous high prevalence, positive resistance	IV	Low
Edwards [23]	AB	98	Retrospective cohort	Isoniazid resistant (Hr-)TB	Foreign-born, Indigenous	Foreign-born positive Hr-TB, Indigenous negative	III	Low
Farzad [24]	Canada	458	Prospective cohort	Culture positive TB	Foreign-born, Indigenous	Foreign-born negative resistance	III	Low
Guthrie [25]	BC	49	Retrospective review	Culture positive TB	Foreign-born	Foreign-born high prevalence, negative resistance	IV	Low
Hersi [26]	AB, BC	4066	Retrospective review	Any TB infection	Foreign-born, Indigenous	Foreign-born positive any resistance, negative MDR	IV	Low
Hirama [27]	Toronto, ON	402	Retrospective cohort	Any TB infection	Foreign-born, homeless	Europe born positive MDR, homeless/refugee status negative resistance	III	Med
Khan [28]	Toronto, ON	91	Retrospective cohort	Any TB infection	Homeless	Homeless negative resistance	III	Med
Lafreniere [30]	Canada	9745	Cross-sectional	Any TB infection	Foreign-born, Indigenous	Foreign-born positive MDR, Indigenous higher prevalence non-resistant	IV	Low
Lafreniere [29]	Canada	1459	Cross-sectional	Any TB infection	Foreign-born	Foreign-born positive any resistance	IV	Low
Langlois-Klassen [31]	AB	1827	Retrospective cohort	Beijing strain TB	Foreign-born	Foreign-born positive Beijing, positive MDR	III	Low
Long [35]	MB	1478	Retrospective cohort	Culture positive TB	Foreign-born, Indigenous	Foreign-born positive any resistance, Indigenous negative	III	Low
Long [33]	AB, BC	768	Cross-sectional	Any TB infection	Foreign-born	Foreign-born positive any resistance	IV	Low
Long [32]	AB, SK	428	Retrospective case–control	Pulmonary TB, FQ use	Indigenous status	Indigenous positive multiple FQ use, negative resistance	III	Low
Long [34]	AB	2234	Retrospective cohort	Culture positive MDR vs non-MDR in foreign born	Foreign-born	Retreatment of TB positive for MDR among foreign-born	III	Low
Marras [36]	Toronto, ON	189	Retrospective review	Culture positive TB	Tibetan refugees	Refugees positive for resistance	IV	Low
Minion [37]	Canada	15,993	Retrospective cohort	Culture positive TB	Foreign-born, Indigenous	Foreign-born positive resistance and MDR, retreatment positive resistance	III	Low
Phongsamart [38]	ON	121	Retrospective review	Any TB infection	Foreign-born	Foreign-born positive resistance	IV	Low
Rivest [39]	Montreal, QC	798	Cross-sectional	Active TB	Foreign-born	Foreign-born high prevalence, negative resistance	IV	Low

*AB:* Alberta; *BC:* British Columbia; *Hr-TB:* isoniazid-resistant tuberculosis; *MB:* Manitoba; *MDR:* multi-drug resistant; *ON:* Ontario; *SK:* Saskatchewan; *TB:* tuberculosis; *QC:* Quebec

**Table 3:** Studies exploring relationship between poverty indicators and ARO-associated acute otitis media

Author and year	Location	Participants	Type of study	Type of infection	Poverty indicator used	Association of resistance with poverty indicator	Quality level (JBI)	Composite rating
Ford-Jones [40]	Toronto, ON	601 children	Prospective cohort	Culture positive acute otitis media	Income, education	Income protective of resistance, maternal education protective	III	High

*ON:* Ontario

**Table 4:** Studies exploring relationship between poverty indicators and invasive bacterial diseases

References	Location	Participants	Type of study	Type of infection	Poverty indicator used	Association of resistance with poverty indicator	Quality level (JBI)	Composite rating
Bocking [41]	Northwestern ON	65	Cross-sectional	Invasive Group A Strep	Indigenous, remote	Indigenous higher prevalence, positive for resistance	IV	Low
Bruce [42]	Alaska, Canada, Finland, Norway, Sweden, Iceland, Greenland	11,244	Cross-sectional	*S. pneumoniae* invasive disease	Indigenous	Indigenous higher prevalence, insufficient data on association with resistance	IV	Low
Cerqueira [43]	Thunder Bay, ON	24	Case series	*H. influenzae* disease	Indigenous	Indigenous higher prevalence, insufficient data on association with resistance	IV	Low
Gounder [44]	North American Arctic, Canada, Alaska, Greenland	247 (74 Canada)	Retrospective review	Bacterial meningitis	Indigenous	Indigenous higher prevalence, insufficient data on association with resistance	IV	Low
Helferty [45]	Northern Canada (NWT, Nunavik, North Labrador, QC)	433	Cross-sectional	*S. pneumoniae* invasive disease	Indigenous, remote	Indigenous higher prevalence, insufficient data on association with resistance	IV	Low

*NWT:* Northwest Territories; *ON:* Ontario; *QC:* Quebec

**Table 5:** Studies exploring relationship between poverty indicators and *H. pylori*

Reference	Location	Participants	Type of study	Type of infection	Poverty indicator used	Association of resistance with poverty indicator	Quality level (JBI)	Composite rating
Morse [46]	Aklavik, NWT	104	RCT standard therapy vs sequential therapy	*H. pylori* colonization	Indigenous, remote	Indigenous higher prevalence, negative for resistance	I	Low

*H. Pylori: Helicobacter pylori; NWT:* Northwest Territories; *RCT:* randomized controlled trial

**Table 6:** Studies exploring relationship between poverty indicators and gonorrhea

Reference	Location	Participants	Type of study	Type of infection	Poverty indicator used	Association of resistance with poverty indicator	Quality level (JBI)	Composite rating
Singh [47]	AB	8535	Cross-sectional	Culture positive gonorrhea	Indigenous	Indigenous negative for resistance and prevalence	IV	Low

*AB:* Alberta

### Methicillin-resistant staphylococcus aureus (MRSA)

Our search yielded 15 articles focused on MRSA (Table 1). One study, a population-based retrospective review of community-acquired MRSA (CA-MRSA) cases in Calgary between 2004 and 2014 by Gill et al. 2019 [10], specifically studied income and risk of MRSA infection. In their retrospective review, which included 4521 cases of CA-MRSA, each additional $100,000 increase in mean household income conferred a 73% relative risk reduction (RR 0.27, 95%CI 0.19–0.39, *p* =  < 0.0001) for MRSA infection amidst a rising population prevalence of CA-MRSA during the study period. The authors argued the protection related to income was a result of lower rates of homelessness and incarceration within this group.

Five of the studies used homelessness as an indicator of poverty [5, 8, 9, 17, 18]. Two of these studies found that homelessness or contact with a shelter was not independently associated with CA-MRSA or polyclonal MRSA infection or colonization [5, 9], while the other three studies did show a relationship between transient residence and either prevalence of CA- or HA-MRSA, or risk of CA- or HA-MRSA as the pathogen compared to methicillin-sensitive *Staphylococcus aureus* [8, 17, 18]. The studies with a positive association were smaller cohorts of participants. Gilbert et al. 2006 [8] who found an association between homelessness and CA-MRSA, did not clearly specify the impact of each poverty measure in their analysis. Participants with a CA-MRSA infection in this study [8] were grouped by a composite measure of risk, with "high-risk" meaning a combination of homelessness, a history of intravenous drug use and past incarceration, thus we are unable to ascribe a higher risk of CA-MRSA infection to homelessness alone. A follow-up study by Gilbert et al. in 2007 [9] sought to better understand the relationship between the prevalence of CA-MRSA infection or colonization and homelessness by exploring several independent risk factors. They evaluated the variables of homelessness and behavioural patterns of drug use among 271 participants. Interestingly, risk factors such as using old antibiotic prescriptions for prior skin infections (OR 4.29, 95% CI 1.07–17.14), having others manipulate your skin (OR 9.55, 95% CI 2.74–33.26), using drugs with a sex worker (OR 5.86, 95% CI 1.63–21.00) or casual partner (OR 5.40, 95% CI 1.64–17.78) or in a hotel (OR 5.14, 95% CI 1.26–20.88), or using drugs many times a day (OR 5.29, 95% CI 1.61–17.41) were all shown to be independently associated with CA-MRSA (among other factors), while homelessness itself was not (OR 0.50, 95% CI 0.16–1.61).

Nine studies examined MRSA in Indigenous peoples [5–7, 11–13, 15, 16, 19]. All studies found a dramatically elevated prevalence of MRSA, particularly CA-MRSA in remote communities, with rates as high as 2,482/100,000 in Northwestern Ontario in 2013 [15]. Several of the authors suggested the elevated rate was a result of crowded living conditions, poor water and housing quality, and high rate of antibiotic use in these communities [5–7, 12, 13, 15, 16, 19]. However, only the study by Daley et al. [5] statistically analyzed socioeconomic risk factors as they related to prevalence of CA-MRSA and polyclonal MRSA colonization. They found the number of rooms in a house to be a significant protective factor (OR 0.86, *p* = 0.023), whereas contact with a homeless shelter (OR 0.60, *p* = 0.63) or correctional facility (OR 1.20, *p* = 0.83) had no effect on CA- or polyclonal MRSA infection rates in multivariate analysis. None of these nine studies directly examined income in their analysis.

### Tuberculosis (TB)

Twenty articles describing the epidemiological risks for TB were included in our study (Table 2). Of these, 19 articles stratified their populations by foreign-born and Canadian born status, emphasizing the importance this variable has on risk of infection with TB. The remaining article examined fluoroquinolone use as it related to resistant TB in Indigenous populations in Alberta and Saskatchewan [32].

These 19 articles similarly describe the burden of TB disease lying within the foreign-born population, whether susceptible, mono-resistant, multi-drug resistant (MDR) or extremely-drug resistant (XDR) TB. Fifteen of 18 articles [20–23, 26, 27, 29–31, 33–38] that looked at the relationship of being foreign-born to having a diagnosis of resistant TB were positively associated, while three found no association [24, 25, 39]. The largest of the studies by Minion et al. in 2013 [37], reported data for 15,993 cases of culture-positive TB across Canada between 1997–2008, of which 66.5% (10,642 cases) were among foreign-born persons, with a 7.7% rate of resistance. They statistically analyzed risk factors for resistance, demonstrating foreign-born persons had 4.3 times the odds (95% CI 2.23–9.69) of MDR-TB compared to Canadian non-Indigenous persons and 1.40 times higher odds (95% CI 1.18–1.67) of resistant non-MDR-TB [37]. Being a re-treatment case of TB was also a statistically significant risk factor of MDR and non-MDR resistant infection (OR 5.79, 95% CI 4.09–8.11, and OR 1.24 95% CI 1.01–1.52, respectively), and resistant cases were more likely to present within 5 years of arrival to Canada compared to susceptible cases. Risk of resistance was positively associated with hailing from Eastern Europe (OR 4.3, 95% CI 1.38–18.92) and the Western Pacific (OR 2.90, 95% CI 1.19–9.20), but not from the African continent, Latin America or Southeast Asia regions [37], further highlighting region of origin is certainly a factor in risk of resistant TB but is not the only factor likely to explain the heterogeneity of risk within these populations. Hypotheses explaining the difference in rates of TB among Canadian and foreign-born persons include standardized, rather than individualized, treatment regimes, lack of susceptibility testing, and lack of second-line treatment options in high-incidence, low-income countries [37].

None of the 20 articles used income as a variable for analysis, nor did the articles explore immigration status, or other measures of marginalization that may be contributing to variable risk. While one article did discuss Tibetan refugee status [36] they did not offer any data on history of living in refugee camps. Regarding Indigenous status and TB, Long et al. 1993 [35] showed no difference in resistance between on-reserve and off-reserve Indigenous groups; and Long et al. in 2009 [32] did not find an association between Indigenous status and resistance.

Two retrospective cohort studies [27, 28] conducted in Toronto examined homelessness and its relationship to resistant TB infection. Both studies had small numbers of participants and found no significant association with homelessness and resistant infection. In fact, the authors suggested that resistant TB in the Toronto homeless was relatively rare, perhaps because many of the visibly homeless persons in Canada are Canadian-born men.

### Acute otitis media

Ford-Jones et al. in 2002 [40] conducted a high-quality, prospective, multi-site cohort study to examine the pathogens in 601 children awaiting myringotomy in Toronto hospitals between 1999 and 2000 (Table 3). They considered definite otitis media pathogens as any aspirate containing one or more of: *H. influenzae, Moraxella catarrhalis, Streptococcus pneumoniae, Staphylococcus aureus,* or group A *Streptococcus* and tested for patterns of antibiotic resistance amongst pathogens*.* They surveyed parents about several sociodemographic risk factors and controlled for these in univariate and multivariate analysis examining prevalence of resistant pathogens. Twenty-one percent of the children had evidence of at least one resistant pathogen. Controlling for several clinical and demographic variables, in univariate analysis the children whose household income was reported as greater than $60,000 had an odds ratio of 0.83 (*p* = 0.039) of having a resistant pathogen, though this was not significant in multivariate analysis. However, each increase in mother’s level of education, defined as completion of some high school, completion of high school or some post-secondary education, was protective from resistant organisms with an odds ratio of 0.68 (95% CI 0.55–0.84, *p* =  < 0.001) in multivariate analysis. These findings were reported alongside more traditional risk factors for acute otitis media in children including smoking and daycare attendance. They noted their population tended to be higher income and higher educated than the general population.

### Other invasive bacterial diseases

Five studies examined invasive bacterial diseases including invasive *H. influenzae, Streptococcus pneumoniae,* group A *Streptococcus* (GAS)*,* and *Neisseria meningitidis* (Table 4)*.*

One study by Bocking and colleagues in 2016 [41] established a link between Indigenous groups in Northern Ontario with a higher rate of resistance than the remainder of Ontario within the same period. Their epidemiological study revealed a tenfold rate of invasive GAS infections among Indigenous people of Northern Ontario compared to non-Indigenous that also had higher rate of erythromycin and clindamycin resistance compared to Ontario in general. The study, however, did not directly compare susceptibility profiles to population demographics of income, though their study population only included remote, First Nations communities.

Bruce et al. 2008 [42] conducted the largest cross-sectional study elucidating the epidemiology of invasive pneumococcal disease (IPD) among circumpolar countries (Alaska, Northern Canada, Finland, Norway, Sweden, Iceland, Greenland) in the pre- and post- 7-valent pneumococcal conjugate (PCV7) vaccination era. These countries are comprised of a high proportion of remote Indigenous peoples. Over the seven-year study, 11,244 cases were examined in the seven countries, where they clearly showed an elevated risk for IPD in Northern Canada of 3.6 times the risk (95% CI 2.6–5.2) for Canadian Indigenous persons compared to non-Indigenous persons. Helferty et al. in 2013 [45] showed a similarly elevated rate of IPD among the Indigenous of in Northern Canada. However, they also did not provide sufficient data on susceptibility testing and intersection with demographic data.

Comparable results were shown in the Gounder et al. 2015 [43] study of bacterial meningitis in the Arctic. Meningitis patients were more likely to be Indigenous than non-Indigenous in Northern Canada and Alaska with a relative risk of 5.2 (95% CI 4.3–6.3) to 1.5 (95% CI 1.2–1.8) respectively. While they performed susceptibility testing, they did not stratify risk of resistance by ethnicity or other poverty indicators.

In contrast, the study by Cerqueira et al. in 2019 [43] found invasive *H. influenzae* to be higher among non-Indigenous people than Indigenous, a relative outlier in the literature of the Arctic populations and invasive bacterial disease. Among the 24 cases of invasive Hemophilus infection presented in this report, there were 4 instances of antibiotic resistance (2 to ampicillin, 2 to trimethoprim-sulfamethoxazole and 1 to cefuroxime), however the data were insufficiently reported to draw conclusions about a link to poverty.

### Helicobacter pylori

Morse et al. 2013 [46] studied *Helicobacter pylori (H. pylori)* infection and its complications in the Arctic, where there has been shown to be a high prevalence of infection (Table 5).

Prior research cited by Morse et al. [46] revealed a connection between risk of infection and lower socioeconomic status, household crowding and familial contacts with infection. However, this study did not specifically control for these factors. They devised a randomized-controlled trial to compare the standard of care for *H. pylori* eradication to sequential, alternative therapy in this population where prevalence rates are as high as 62% compared to less than 30% in an average, non-Arctic population.

They found poor treatment success with both regimens; however, a slight advantage was noted with the newer, sequential therapy. Thirteen of 89 participants were infected with resistant *H. pylori*, either to metronidazole or clarithromycin. They did not randomize the participants based on antimicrobial susceptibilities. They reported the frequency of resistance in the Aklavik population to be similar to the rest of Canada, and the clarithromycin resistance rate of 8% was lower than the reported Canadian rate of 15%.

Overall, there is a high burden of *H. pylori* infections and related complications in the Canadian North, and although treatment outcomes were less successful, this did not appear to be a result of antibiotic resistance, as similar rates of resistance were reported. Further, being located in Aklavik was not associated with an increased rate of resistant *H. pylori* infection. There is not currently enough evidence to suggest that resistance patterns among *H. pylori* are related to low income despite the elevated burden of disease.

### Gonorrhea

One study by Singh and colleagues from 2013 [47] examined gonococcal isolates in Alberta from 2007 to 2011, looking at resistance patterns in various demographic groups during this time (Table 6).

They noted a small but significant rise in the proportion of isolates resistant to penicillin and tetracyclines in 2010. However, this increase seemed mainly linked to a cluster of isolates associated with men who have sex with men. Indigenous groups did not have an elevated rate of resistance. Overall, this is a low-quality study for our question of interest that did not strongly link resistant infections to a poverty indicator.

## Discussion

Our narrative review has revealed an epidemiological link between the dimensions of poverty and infection with an ARO. These studies have demonstrated that, with respect to organisms including MRSA, predominantly CA-MRSA, TB, certain invasive bacterial diseases, organisms associated with acute otitis media, *H. pylori* and *N. gonorrhoeae*, there are social factors that influence rates of disease, however, as it relates to income, we found data to be rather limited and generally of low quality.

It is worth noting that risks of the burden of infectious diseases in general correlates with the risks of acquiring an ARO infection. The relationship between poverty and infectious disease is well described in the literature, for example, the Global Burden of Disease studies, as the broadest example of this phenomenon [56]. In the United States, Gohil [57] found that all-cause burden of infection, and hospital readmissions related to infections were highest among the populations in federal poverty areas. In turn, our review also suggests there is an independent relationship between the dimensions of poverty and ARO infections, as evidenced by our findings among invasive bacterial diseases, where there were high rates of infections without findings of high rates of resistance [42–45].

Regarding income specifically, we found only two available studies in the literature that addressed the relationship to infections with ARO infections directly. Ford-Jones et al. 2002 [40] looked at otitis media and associated ARO-associated infections (high quality), and Gill et al. 2019 [10] (medium quality) with CA-MRSA, establishing a statistical association between higher income and lower risk of ARO infections. These two articles are higher quality studies that support a protective impact of income against resistant organism infections, supporting an association for ARO-associated otitis media and CA-MRSA.

The evidence base for poverty indicators and the link to resistant organisms was most strongly established for CA-MRSA, though evidence exists for HA-MRSA as well, in our review. Our study found six medium-quality [5, 8–10, 17, 18] and nine low-quality [6, 7, 11–16, 19] articles lending to our question of interest. Of the six medium quality articles, five related to homelessness (3 with a positive association) [5, 8, 9, 17, 18], one to income [10] and one to Indigenous status [5]. It is reasonable to conclude from these studies that there is a positive association between CA-MRSA, and HA-MRSA to a lesser extent, and homelessness [8, 17, 18], though, as outlined by Gilbert et al. 2007 [9], significant variables associated with CA-MRSA were behaviours such as frequent drug use, using drugs with casual sexual contacts, and having others manipulate one’s skin [9], whereas contact with a shelter, group-living facility or jail was not shown to be associated. This study helps unpack the more nuanced relationship that likely exists between homelessness and transmission of MRSA, suggesting that behavioural patterns associated with homelessness are confounding this relationship. We can also conclude from the data that there is an elevated prevalence and incidence of predominantly CA-MRSA in Indigenous groups [5–7, 11–13, 15, 16], though the evidence is relatively weak on the aspects of living conditions contributing to this effect.

On the topic of tuberculosis, we found 18 low quality [20–26, 29–39] and two medium-quality studies [27, 28]. The low-quality studies provided an epidemiological synthesis of TB in Canada, which show an overwhelmingly positive relationship between being foreign-born and an increased prevalence of TB and an increased risk of resistance. Foreign-born persons carry the burden of disease of TB in Canada regardless of susceptibility, with established risk factors for resistant infections being prior treatment for TB and more recent arrival to Canada [27, 34, 37]. None of the studies, however, provide deeper analysis of the socioeconomic factors associated with being foreign-born that may be contributing to this pattern of resistance. If we accept the premise that being foreign-born comes with the heightened risk of poverty due to social barriers to health experienced by many immigrants, it would be reasonable to conclude that poverty and resistant TB are likely associated, but the data do not substantiate this. This lack of understanding of the economic and social factors that influencing the development of drug resistance in foreign-born persons hinders our ability to intervene. Both medium quality articles [27, 28] looking at drug-resistant TB and homelessness did not find any association. None of the 20 studies used income in their analysis, thus we cannot conclude with certainly that income confers protection from resistant TB.

There were five articles of low-quality evidence that reviewed invasive bacterial diseases in Northern Canada [41–45]. While rates of these diseases are elevated among Indigenous groups of the Canadian North, no association can be made between poverty and resistant invasive bacterial disease. One study by Bocking et al. [41] did show an elevated prevalence of erythromycin and clindamycin-resistant group A *Streptococcal* infection (24.6% for both) in the Indigenous community during the study period, however, susceptibility profiles were not directly compared to demographic factors in the population, making this weak evidence for our question of interest.

The remaining four studies of invasive bacterial disease [42–45], and one study regarding *H. pylori* [46] did not find an association with ARO infections and Indigenous status. Many of these authors posited that crowding, environmental stress, rates of substance use, and lower socioeconomic status were ‘causal’ factors in the disparity of invasive bacterial diseases and *H. pylori* among Indigenous peoples of the North [41, 42, 44–46], however, these socioeconomic factors were not controlled for in these studies. While epidemiologic data is clear regarding the elevated burden of disease that exists for invasive bacterial diseases and *H. pylori* in the Canadian North among Indigenous communities, the data are insufficient to draw conclusions between poverty and increased risk of resistance in these infections. Similarly, in the USA, Chen et al. in 2018 found that rates of susceptible invasive *Streptococcus pneumoniae* infections were higher among residents of lower median household income areas, but that penicillin non-susceptible strains of *S. pneumoniae* were elevated in higher-income residents, theorized to be due to increased used of antimicrobials among those with higher income [51].

At this time, little is known about any relationship between antimicrobial resistant gonorrhea or other sexually transmitted infections and income status. While many factors are implicated in the discussion of resistant gonorrhea, one study in China [49] examined socioeconomic status and resistant gonorrhea infections showing the middle-income demographic had lower rates of plasmid-mediated resistance to tetracyclines compared to the lower-income bracket.

To our knowledge there is only one other published review related to dimensions of poverty and antimicrobial resistance, which was conducted by Alividza and colleagues in 2018 [2], and is international in scope. Similar to our findings, their review demonstrated low-income, homelessness, drug-use and poor housing quality were risk factors for MRSA in the USA. One study included in their review linked social deprivation to higher rates of MRSA in the UK [55]. An Indonesian study also found low-income to be a risk factor for MRSA carriage [50]. Alivizda et al. [2] highlighted the international paucity of evidence specific to income and antimicrobial resistance, finding only four such articles, and our review finding two articles. It is noteworthy that many people experiencing poverty avoid seeking health care due to both real and perceived barriers to care, including the cost of treatment, systemic bias within the health care system, or inadequate accessibility. These barriers to accessing care mean antimicrobial resistance may be underrecognized in these communities.

In reviewing the greater body of literature related to poverty and antimicrobial resistance, the discussion largely focuses on low- and middle-income countries as poverty affects a higher proportion of the population, alongside a higher burden of infectious diseases [52]. Out-of-pocket health care expenses, suboptimal treatment regimens, lack of diagnostic testing, sharing medications, use of low-quality or expired medications, for-profit providers and pharmacies with loose regulations all create a propensity towards antimicrobial resistance that disproportionately affects the poor [53, 54].

A global narrative or systematic review of our question of interest would certainly enhance our knowledge base and understanding of the granularity and complexity of the ties between patterns of antimicrobial use and resistance in impoverished populations.

## Strengths and limitations

A strength of our structured narrative review is that it is highly replicable with transparent methodology. We have outlined the importance of this study and its contribution to the medical and public health literature and it is one of the first of its kind from a Canadian perspective. We had pre-defined inclusion and exclusion criteria. We have made our methodology, and quality assessment explicit, and all the data used for our synthesis is available in tables and references within the manuscript. Overall, our review scores highly on the SANRA – the Scale for the Assessment of Narrative Review Articles [48], a quality appraisal tool for narrative review studies.

We acknowledge that our review has limitations. Given the observational epidemiological design used in most articles included in our review, it is important to emphasize that while our data appear to support an association of certain individual dimensions of poverty and rates of infection with AROs, we cannot conclude that these demographic factors are indeed causal factors leading to an increased rate of resistant infection. Similarly, we were unable to calculate statistical measures of ‘risk’ with our methodology, and instead have presented the available literature that suggests an association between poverty and ARO infections.

Ideally, we would have uncovered broader population-based studies that would allow us to examine varying rates of infection between income groups, rather than study samples selected for infection without comparison groups. It is possible we may be seeing the effect of an increase in infections in general within impoverished population groups. Furthermore, a qualitative research perspective is also lacking in the current knowledge base and will be needed to understand how experiences of poverty impact ARO infections through effects on access to healthcare, care seeking and health system engagement. Without broader population-based studies and a qualitative understanding, validation of causality remains limited.

Our study is subject to the selection biases inherent in any narrative review. However, we attempted to mitigate this bias by including three scientific databases using a systematic search process, nonetheless we cannot ensure all possible relevant research was included in our review based on the limitations inherent in a database search development, title and abstract and screening process. We also acknowledge the possibility of subjectivity within assigning and interpreting the quality of the included studies, but we worked to minimize subjectivity using a rigorous approach to quality assessment. We adhered to a consistent application of the Joanna Briggs Institute Levels of Evidence [4] framework, along with development and application of standardized criteria for assessing the strength of the poverty indicator, which were reviewed independently by the authors.

## Conclusion

Our structured narrative review is the first of its kind in Canada and contributes to our understanding of the relationship between poverty and infections with AROs from a Canadian perspective. There is evidence that higher income is associated with protection from CA-MRSA infection and ARO-associated acute otitis media in children. Mixed results exist outlining the relationship between homelessness and MRSA, demonstrating a more nuanced association relating to behavioural risk factors as an underlying driver. We do not yet have a robust understanding of the socioeconomic aspects of being foreign-born that contribute to higher rates of resistant TB infection in Canada. There is an elevated burden of disease for invasive bacterial diseases and *H. pylori* in the Canadian North among Indigenous communities, yet the data are insufficient to link poverty and risk of resistance. Data on resistant sexually transmitted infections and poverty is limited at this time.

Untangling the ways that social and material deprivation influence downstream clinical presentations is complex, but necessary. Based on our findings, we would suggest researchers include income as a variable of interest in scientific analysis when examining antimicrobial resistant infections. Considering the impact of poverty and its dimensions on health outcomes will allow clinicians, researchers and policymakers to better address the complex issue of antimicrobial resistance and vulnerability in Canada.

## Supplementary Information

**Additional file 1.** Detailed Search Strategy

**Additional file 2.** Detailed Grey Literature Methodology

## Data Availability

All data generated or analyzed during this study are included in this published article [and its supplementary information files].

## Abbreviations

AB: Alberta
AMR: Antimicrobial resistant (resistance)
ARO: Antimicrobial resistant organism(s)
BC: British Columbia
CA-MRSA: Community-acquired Methicillin-resistant *Staphylococcus aureus*
GAS: Group A *Streptococcus*
HA-MRSA: Hospital-acquired Methicillin-resistant *Staphylococcus aureus*
*H. influenzae*: *Haemophilus influenza*
*H. pylori*: *Helicobacter pylori*
Hr-TB: Isoniazid-resistant tuberculosis
IPD: Invasive pneumococcal disease
JBI: Joanna-Briggs Institute
MB: Manitoba
MDR: Multi-drug resistant
NL: Newfoundland
NWT: Northwest Territories
ON: Ontario
PCV-7: 7-Valent pneumococcal conjugate vaccine
PRISMA: Preferred Reporting Items for Systematic Reviews and Meta-Analyses
QC: Quebec
RCT: Randomized controlled trial
SANRA: Scale for the quality assessment of narrative review articles
SK: Saskatchewan
TB: Tuberculosis
USA: United States of America
XDR: Extremely drug resistant
