# Supplementary material for: Dimensions of poverty as risk factors for antimicrobial resistant organisms in Canada: a structured narrative review
Source: Antimicrob Resist Infect Control. 2022 Jan 24;11:18. doi: 10.1186/s13756-022-01059-1 (PMC8785485; doi:10.1186/s13756-022-01059-1)
Supplement: Supplementary file 1 — Additional file 1. Detailed Search Strategy [file 13756_2022_1059_MOESM1_ESM.docx]

**Additional File 1: Detailed Peer-Reviewed Search Strategy**

**Supplemental Document:**

King T, et al: Dimensions of poverty as risk factors for antimicrobial resistant organisms in Canada: A structured narrative review.

**MedLINE SEARCH**

**Performed September 27, 2020**

1 Poverty/ (38272)
2 Poverty Areas/ (6114)
3 poverty.mp. (60496)
4 ((low* or bottom or strain) adj2 (income or status or financ*)).mp. (63922)
5 socioeconomic factors/ or economic status/ or educational status/ or employment/ or income/ or social class/
(280769)
6 academic failure/ or literacy/ (830)
7 (socioeconomic factors or economic status or educational status or income or social class or employment or
academic failure or literacy).mp. (436563)
8 ((food or hous*) adj2 (insecur* or unmet)).mp. (4561)
9 indians, north american/ or inuits/ (17222)
10 (native american* or inuit* or aborigin* or indigenous or first nation* or metis).mp. (54330)
11 (reserve* or reservation*).mp. (85527)
12 homeless persons/ or homeless youth/ or vulnerable populations/ (19501)
13 (homeless* or ((homeless* or street or vulnerable) adj2 (people or person* or kid or kids or youth* or child* or
population*))).mp. (37609)
14 "emigrants and immigrants"/ or undocumented immigrants/ or medically uninsured/ or refugees/ or "transients and
migrants"/ or working poor/ (40018)
15 (refugee* or transient* or migrant* or asylum seek* or immigrant* or ((undocument* or unauthori* or illegal) adj2
(immigrant* or alien* or worker*)) or ((underinsured or uninsured) adj2 (medically or person* or people))).mp. (412764)
16 1 or 2 or 3 or 4 or 5 or 6 or 7 or 8 or 9 or 10 or 11 or 12 or 13 or 14 or 15 (1035494)
17 drug resistance.mp. (272010)
18 drug resistance/ or drug resistance, microbial/ or drug resistance, bacterial/ or beta-lactam resistance/ or
cephalosporin resistance/ or penicillin resistance/ or ampicillin resistance/ or methicillin resistance/ or
chloramphenicol resistance/ or drug resistance, multiple, bacterial/ or kanamycin resistance/ or tetracycline
resistance/ or trimethoprim resistance/ or vancomycin resistance/ or drug resistance, multiple/ (203029)
19 (resistan* adj3 (organism* or bacteria* or microbe* or microbial* or antimicrobial or antibacteria* or
anti-bacteria* or antibiotic* or drug)).mp. (350241)
20 ((penicillin or amoxicillin or ampicillin or oxacillin or nafcillin or dapsone or amikacin or gentamicin or
tobramycin or streptomycin or azithromycin or ertapenem or meropenem or imipenem or cef* or ceph* or ciprofloxacin or
moxifloxacin or levofloxacin or gemiflox* or tigecycline or clindamycin or daptomycin or clarithromycin or erythromycin
or fidaxomicin or aztreonam or nitro* or metronidazole or linezolid or colistin or rifa* or beta-lactam or
chloramphenicol or doxycycline or tetracycline or trimethoprim or kanamycin or vancomycin) adj2 resistan*).mp. (75110)
21 Methicillin-Resistant Staphylococcus aureus/ (14673)
22 (Methicillin-Resistant Staphylococcus aureus or MRSA).mp. (33376)
23 ((aminoglycoside or carbapenem or cephalosporin or fluoroquinolone or glyc* or macrolide or monobactam or
penicillin or polymyxin or tetracycline) adj3 resistan*).mp. (43521)
24 ((pseudomonas or enterobact* or enterococc* or helicobacter or campylobacter or salmonella* or neisseria* or
streptococc* or haemophil* or acinetobacter or tuberculosis or staphylococcus) adj3 resistan*).mp. (78234)
25 (extended spectrum beta-lactamase or ESBL or ampc).mp. (12628)
26 17 or 18 or 19 or 20 or 21 or 22 or 23 or 24 or 25 (417935)
27 exp Canada/ (159667)
28 (canad* or alberta or british columbia or saskatchewan or manitoba or ontario or quebec or nova scotia or new
brunswick or newfoundland or labrador or prince edward island or yukon or nunavut or northwest territories or
nunavik).mp. (239296)
29 27 or 28 (239296)
30 16 and 26 and 29 (168)
31 limit 30 to last 30 years (155)

**EMBASE SEARCH**

**September 27, 2020**

1 poverty.tw,kw. or exp poverty/ (57641)
2 ((low* or bottom or strain) adj2 (income or status or financ*)).tw,kw. (78187)
3 income/ or income group/ or social status/ or socioeconomics/ or lowest income group/ or economic status/ or
household economic status/ or social class/ (315207)
4 academic achievement/ or achievement/ or education/ or academic failure/ or academic underachievement/ or
literacy/ (478847)
5 employment status/ or unemployment/ (34036)
6 indigenous people/ or canadian aboriginal/ or first nation/ (7308)
7 (lowest income group or income group or employment status or socioeconomics or socioeconomic factors or economic
status or household economic status or social status or educational status or income or social class or employment or
unemployment or academic failure or academic underachievement or literacy).tw,kw. (291203)
8 food insecurity/ (3352)
9 ((food or hous*) adj2 (insecur* or unmet)).tw,kw. (5762)
10 (native american* or inuit* or aborigin* or indigenous or first nation* or metis).tw,kw. (61847)
11 (reserve* or reservation*).tw,kw. (1394754)
12 homeless man/ or homeless person/ or homeless woman/ or homeless youth/ (2181)
13 vulnerable population/ (17860)
14 (homeless* or ((homeless* or street or vulnerable) adj2 (people or person* or kid or kids or youth* or child* or
population*))).tw,kw. (34483)
15 immigrant/ or migrant/ (24330)
16 undocumented immigrant/ (456)
17 working poor/ or lowest income group/ (28273)
18 refugee/ or asylum seeker/ (13366)
19 (refugee* or migrant* or asylum seek* or immigrant* or working poor or (low* income adj2 group*) or ((undocument*
or unauthori* or illegal) adj2 (immigrant* or alien* or worker*))).tw,kw. (59284)
20 1 or 2 or 3 or 4 or 5 or 6 or 7 or 8 or 9 or 10 or 11 or 12 or 13 or 14 or 15 or 16 or 17 or 18 or 19 (2466793)
21 drug resistance/ or antibiotic resistance/ or antimalarial drug resistance/ or extensive drug resistance/ or
multidrug resistance/ (281925)
22 antibiotic resistance/ or aminoglycoside resistance/ or beta-lactam resistance/ or chloramphenicol resistance/ or
daptomycin resistance/ or fluoroquinolone resistance/ or macrolide resistance/ or polymyxin resistance/ or rifampicin
resistance/ or tetracycline resistance/ or trimethoprim resistance/ or vancomycin resistance/ (159493)
23 amikacin resistance/ or gentamicin resistance/ or kanamycin resistance/ or streptomycin resistance/ or tobramycin
resistance/ or cefotaxime resistance/ or ceftazidime resistance/ or ciprofloxacin resistance/ or erythromycin
resistance/ or polymyxin resistance/ or colistin resistance/ (1314)
24 drug resistance.tw,kw. (88723)
25 (resistan* adj3 (organism* or bacteria* or microbe* or microbial* or antibacteria* or anti-bacteria* or
antimicrobial or antibiotic* or drug)).tw,kw. (249179)
26 antimicrobial resistan*.tw,kw. (30165)
27 ((penicillin or amoxicillin or ampicillin or oxacillin or nafcillin or dapsone or amikacin or gentamicin or
tobramycin or streptomycin or azithromycin or ertapenem or meropenem or imipenem or cef* or ceph* or ciprofloxacin or
moxifloxacin or levofloxacin or gemiflox* or tigecycline or clindamycin or daptomycin or clarithromycin or erythromycin
or fidaxomicin or aztreonam or nitro* or metronidazole or linezolid or colistin or rifa* or beta-lactam or
chloramphenicol or doxycycline or tetracycline or trimethoprim or kanamycin or vancomycin) adj2 resistan*).tw,kw.
(74419)
28 methicillin resistant staphylococcus aureus/ or methicillin resistant staphylococcus aureus infection/ (50858)
29 (methicillin resistant staphylococcus aureus or (methicillin resistant staphylococcus aureus adj2
infection)).tw,kw. (28179)
30 ((aminoglycoside or carbapenem or cephalosporin or fluoroquinolone or glyc* or macrolide or monobactam or
penicillin or polymyxin or tetracycline) adj3 resistan*).tw,kw. (41043)
31 ((pseudomonas or enterobact* or enterococc* or helicobacter or campylobacter or salmonella* or neisseria* or
streptococc* or haemophil* or tuberculosis or acinetobacter or staphylococcus) adj3 resistan*).tw,kw. (86447)
32 extended spectrum beta lactamase/ or extended spectrum beta lactamase producing enterobacteriaceae/ (10577)
33 (extended spectrum beta lactamase or ESBL or extended spectrum beta lactamase producing enterobact*).tw,kw.
(14945)
34 21 or 22 or 23 or 24 or 25 or 26 or 27 or 28 or 29 or 30 or 31 or 32 or 33 (474838)
35 canada/ or alberta/ or british columbia/ or manitoba/ or new brunswick/ or "newfoundland and labrador"/ or
northwest territories/ or nova scotia/ or nunavut/ or ontario/ or prince edward island/ or quebec/ or saskatchewan/ or
yukon/ (183693)
36 (canad* or alberta or british columbia or saskatchewan or manitoba or ontario or quebec or nova scotia or new
brunswick or newfoundland or labrador or prince edward island or yukon or nunavut or northwest territories or
nunavik).tw,kw. (230983)
37 35 or 36 (286972)
38 20 and 34 and 37 (537)
39 limit 38 to last 30 years (527)

**Web of Science SEARCH**

**Performed September 27, 2020**

| # 20 | [**454**](https://apps.webofknowledge.com/summary.do?product=WOS&doc=1&qid=71&SID=8AlF2E4GoDhMiCA48JH&search_mode=AdvancedSearch&update_back2search_link_param=yes) | #15 AND #18 AND #19  *Indexes=SCI-EXPANDED, SSCI, A&HCI, CPCI-S, CPCI-SSH, ESCI Timespan=1990-2020* |  |
| --- | --- | --- | --- |
| # 19 | [**316,894**](https://apps.webofknowledge.com/summary.do?product=WOS&doc=1&qid=70&SID=8AlF2E4GoDhMiCA48JH&search_mode=AdvancedSearch&update_back2search_link_param=yes) | #14 OR #13 OR #12 OR #11 OR #10 OR #9 OR #8  *Indexes=SCI-EXPANDED, SSCI, A&HCI, CPCI-S, CPCI-SSH, ESCI Timespan=1990-2020* |  |
| # 18 | [**6,016,333**](https://apps.webofknowledge.com/summary.do?product=WOS&doc=1&qid=69&SID=8AlF2E4GoDhMiCA48JH&search_mode=AdvancedSearch&update_back2search_link_param=yes) | #17 OR #16 OR #7 OR #6 OR #5 OR #4 OR #3 OR #2 OR #1  *Indexes=SCI-EXPANDED, SSCI, A&HCI, CPCI-S, CPCI-SSH, ESCI Timespan=1990-2020* |  |
| # 17 | [**9,004**](https://apps.webofknowledge.com/summary.do?product=WOS&doc=1&qid=68&SID=8AlF2E4GoDhMiCA48JH&search_mode=AdvancedSearch&update_back2search_link_param=yes) | TS=((food or hous*) NEAR/2 (insecur* or unmet) )  *Indexes=SCI-EXPANDED, SSCI, A&HCI, CPCI-S, CPCI-SSH, ESCI Timespan=1990-2020* |  |
| # 16 | [**700,142**](https://apps.webofknowledge.com/summary.do?product=WOS&doc=1&qid=67&SID=8AlF2E4GoDhMiCA48JH&search_mode=AdvancedSearch&update_back2search_link_param=yes) | TS=(lowest income group OR income group OR employment status OR socioeconomics OR socioeconomic factors OR economic status OR household economic status OR social status OR educational status OR income OR social class OR employment OR unemployment OR academic failure OR academic underachievement OR literacy)  *Indexes=SCI-EXPANDED, SSCI, A&HCI, CPCI-S, CPCI-SSH, ESCI Timespan=1990-2020* |  |
| # 15 | [**379,069**](https://apps.webofknowledge.com/summary.do?product=WOS&doc=1&qid=66&SID=8AlF2E4GoDhMiCA48JH&search_mode=AdvancedSearch&update_back2search_link_param=yes) | TS=(canad* or alberta or british columbia or saskatchewan or manitoba or ontario or quebec or nova scotia or new brunswick or newfoundland or labrador or prince edward island or yukon or nunavut or northwest territories or nunavik)  *Indexes=SCI-EXPANDED, SSCI, A&HCI, CPCI-S, CPCI-SSH, ESCI Timespan=1990-2020* |  |
| # 14 | [**13,494**](https://apps.webofknowledge.com/summary.do?product=WOS&doc=1&qid=65&SID=8AlF2E4GoDhMiCA48JH&search_mode=AdvancedSearch&update_back2search_link_param=yes) | TS=(extended spectrum beta-lactamase or ESBL or ampc)  *Indexes=SCI-EXPANDED, SSCI, A&HCI, CPCI-S, CPCI-SSH, ESCI Timespan=1990-2020* |  |
| # 13 | [**85,213**](https://apps.webofknowledge.com/summary.do?product=WOS&doc=1&qid=64&SID=8AlF2E4GoDhMiCA48JH&search_mode=AdvancedSearch&update_back2search_link_param=yes) | TS=((pseudomonas or enterobact* or enterococc* or helicobacter or campylobacter or salmonella* or neisseria* or streptococc* or haemophil* or acinetobacter or tuberculosis or staph*) NEAR/3 resistan*)  *Indexes=SCI-EXPANDED, SSCI, A&HCI, CPCI-S, CPCI-SSH, ESCI Timespan=1990-2020* |  |
| # 12 | [**41,114**](https://apps.webofknowledge.com/summary.do?product=WOS&doc=1&qid=63&SID=8AlF2E4GoDhMiCA48JH&search_mode=AdvancedSearch&update_back2search_link_param=yes) | TS=((aminoglycoside or carbapenem or cephalosporin or fluoroquinolone or glyc* or macrolide or monobactam or penicillin or polymyxin or tetracycline) NEAR/3 resistan*)  *Indexes=SCI-EXPANDED, SSCI, A&HCI, CPCI-S, CPCI-SSH, ESCI Timespan=1990-2020* |  |
| # 11 | [**36,379**](https://apps.webofknowledge.com/summary.do?product=WOS&doc=1&qid=62&SID=8AlF2E4GoDhMiCA48JH&search_mode=AdvancedSearch&update_back2search_link_param=yes) | TS=(Methicillin-Resistant Staphylococcus aureus or MRSA)  *Indexes=SCI-EXPANDED, SSCI, A&HCI, CPCI-S, CPCI-SSH, ESCI Timespan=1990-2020* |  |
| # 10 | [**64,265**](https://apps.webofknowledge.com/summary.do?product=WOS&doc=1&qid=61&SID=8AlF2E4GoDhMiCA48JH&search_mode=AdvancedSearch&update_back2search_link_param=yes) | TS=((penicillin or amoxicillin or ampicillin or oxacillin or nafcillin or dapsone or amikacin or gentamicin or tobramycin or streptomycin or azithromycin or ertapenem or meropenem or imipenem or cef* or ceph* or ciprofloxacin or moxifloxacin or levofloxacin or gemiflox* or tigecycline or clindamycin or daptomycin or clarithromycin or erythromycin or fidaxomicin or aztreonam or nitro* or metronidazole or linezolid or colistin or rifa* or beta-lactam or chloramphenicol or doxycycline or tetracycline or trimethoprim or kanamycin or vancomycin) NEAR/2 resistan*)  *Indexes=SCI-EXPANDED, SSCI, A&HCI, CPCI-S, CPCI-SSH, ESCI Timespan=1990-2020* |  |
| # 9 | [**234,459**](https://apps.webofknowledge.com/summary.do?product=WOS&doc=1&qid=60&SID=8AlF2E4GoDhMiCA48JH&search_mode=AdvancedSearch&update_back2search_link_param=yes) | TS=(resistan* NEAR/3 (organism* or bacteria* or microbe* or microbial* or antimicrobial or antibacteria* or anti-bacteria* or antibiotic* or drug) )  *Indexes=SCI-EXPANDED, SSCI, A&HCI, CPCI-S, CPCI-SSH, ESCI Timespan=1990-2020* |  |
| # 8 | [**83,824**](https://apps.webofknowledge.com/summary.do?product=WOS&doc=1&qid=59&SID=8AlF2E4GoDhMiCA48JH&search_mode=AdvancedSearch&update_back2search_link_param=yes) | TS=(resistan* NEAR/2 (microbial OR bacterial OR beta-lactam OR cephalosporin OR penicillin OR ampicillin OR methicillin OR chloramphenicol OR multiple-drug OR kanamycin OR tetracycline OR trimethoprim OR vancomycin) )  *Indexes=SCI-EXPANDED, SSCI, A&HCI, CPCI-S, CPCI-SSH, ESCI Timespan=1990-2020* |  |
| # 7 | [**5,883**](https://apps.webofknowledge.com/summary.do?product=WOS&doc=1&qid=58&SID=8AlF2E4GoDhMiCA48JH&search_mode=AdvancedSearch&update_back2search_link_param=yes) | TS=(poor NEAR/2 (person* or people) )  *Indexes=SCI-EXPANDED, SSCI, A&HCI, CPCI-S, CPCI-SSH, ESCI Timespan=1990-2020* |  |
| # 6 | [**211,158**](https://apps.webofknowledge.com/summary.do?product=WOS&doc=1&qid=57&SID=8AlF2E4GoDhMiCA48JH&search_mode=AdvancedSearch&update_back2search_link_param=yes) | TS=(refugee* or migrant* or asylum seek* or immigrant* or working poor or (low* income NEAR/2 group*) or ((undocument* or unauthori* or illegal) NEAR/2 (immigrant* or alien* or worker*) ))  *Indexes=SCI-EXPANDED, SSCI, A&HCI, CPCI-S, CPCI-SSH, ESCI Timespan=1990-2020* |  |
| # 5 | [**40,563**](https://apps.webofknowledge.com/summary.do?product=WOS&doc=1&qid=56&SID=8AlF2E4GoDhMiCA48JH&search_mode=AdvancedSearch&update_back2search_link_param=yes) | TS=(homeless* or ((homeless* or street or vulnerable) NEAR/2 (people or person* or kid or kids or youth* or child* or population*) ))  *Indexes=SCI-EXPANDED, SSCI, A&HCI, CPCI-S, CPCI-SSH, ESCI Timespan=1990-2020* |  |
| # 4 | [**4,903,087**](https://apps.webofknowledge.com/summary.do?product=WOS&doc=1&qid=55&SID=8AlF2E4GoDhMiCA48JH&search_mode=AdvancedSearch&update_back2search_link_param=yes) | TS=(reserve* or reservation*)  *Indexes=SCI-EXPANDED, SSCI, A&HCI, CPCI-S, CPCI-SSH, ESCI Timespan=1990-2020* |  |
| # 3 | [**284,346**](https://apps.webofknowledge.com/summary.do?product=WOS&doc=1&qid=54&SID=8AlF2E4GoDhMiCA48JH&search_mode=AdvancedSearch&update_back2search_link_param=yes) | TS=((native american* OR inuit* OR aborigin* OR indigenous OR first nation* OR metis))  *Indexes=SCI-EXPANDED, SSCI, A&HCI, CPCI-S, CPCI-SSH, ESCI Timespan=1990-2020* |  |
| # 2 | [**114,966**](https://apps.webofknowledge.com/summary.do?product=WOS&doc=1&qid=53&SID=8AlF2E4GoDhMiCA48JH&search_mode=AdvancedSearch&update_back2search_link_param=yes) | TS=((low* or bottom or strain) NEAR/2 ( income OR status OR financ*) )  *Indexes=SCI-EXPANDED, SSCI, A&HCI, CPCI-S, CPCI-SSH, ESCI Timespan=1990-2020* |  |
| # 1 | [**79,670**](https://apps.webofknowledge.com/summary.do?product=WOS&doc=1&qid=52&SID=8AlF2E4GoDhMiCA48JH&search_mode=AdvancedSearch&update_back2search_link_param=yes) | TS=poverty  *Indexes=SCI-EXPANDED, SSCI, A&HCI, CPCI-S, CPCI-SSH, ESCI Timespan=1990-2020* |  |
