# Supplementary material for: Dimensions of poverty as risk factors for antimicrobial resistant organisms in Canada: a structured narrative review
Source: Antimicrob Resist Infect Control. 2022 Jan 24;11:18. doi: 10.1186/s13756-022-01059-1 (PMC8785485; doi:10.1186/s13756-022-01059-1)
Supplement: Supplementary file 2 — Additional file 2. Detailed Grey Literature Methodology [file 13756_2022_1059_MOESM2_ESM.docx]

**Additional File 2 – Detailed Grey Literature Methodology**

**Supplemental Document:**

King T, et al: Dimensions of poverty as risk factors for antimicrobial resistant organisms in Canada: A structured narrative review.

SEARCH TERMS: antimicrobial resistance, antibiotic resistance AND poverty (résistance aux antibiotiques ET pauvreté)

|  | **Organization** | **Reviewed** | **Findings** | **Reason for Exclusion** |
| --- | --- | --- | --- | --- |
| **Federal** | | | | |
| House of Commons |  | Yes | House of Commons Report on AMR Status, Casey 2018 | No original data |
| CNISP |  | Yes | CNISP Report 2017 | No original data |
| CIHI |  | Yes | None | N/A |
| PHAC |  | Yes | Pan-Canadian Framework for Action, 2017: <https://www.canada.ca/content/dam/hc-sc/documents/services/publications/drugs-health-products/tackling-antimicrobial-resistance-use-pan-canadian-framework-action/tackling-antimicrobial-resistance-use-pan-canadian-framework-action.pdf> | No original data |
| Health Canada |  | Yes | TB Standards Report | No original data |
| CIHR |  | Yes | None | N/A |
| **Provincial Health Agencies and Health Authorities** | | | | |
| BC | BC Ministry of Health | Yes | None | N/A |
|  | BC Centre for Disease Control | Yes | AMR Summary Report 2014 | No mention of poverty |
|  | Fraser Health | Yes | None | N/A |
|  | Interior Health | Yes | None | N/A |
|  | Northern Health | Yes | None | N/A |
|  | Vancouver Coastal Health | Yes | None | N/A |
|  | Provincial Health Services Authority | Yes | None | N/A |
|  | Vancouver Island Health Authority | Yes | None | N/A |
| Alberta | Alberta Health | Yes | None | N/A |
|  | Alberta Health Services | Yes | None | N/A |
| Saskatchewan | Saskatchewan Health | Yes | None | N/A |
| Manitoba | Manitoba Health | Yes | None | N/A |
| Ontario | Ontario Ministry of Health and Long-Term Care | Yes | None | N/A |
|  | Public Health Ontario | Yes | None | N/A |
| Quebec | Institut national de santé publique du Québec | Yes | None | N/A |
|  | Quebec Ministry of Health and Social Services | Yes | None | N/A |
| Nunavik | Nunavik Regional Board of Health and Social Services | Yes | None | N/A |
| New Brunswick | New Brunswick Health | Yes | None | N/A |
| Nova Scotia | Nova Scotia Department of Health and Wellness | Yes | None | N/A |
| Newfoundland and Labrador | Newfoundland and Labrador Department of Health and Community Services | Yes | None | N/A |
| PEI | Prince Edward Island Department of Health and Wellness | Yes | None | N/A |
| Northwest Territories | Northwest Territories Health and Social Services | Yes | None | N/A |
| Yukon | Yukon Health and Social Services | Yes | None | N/A |
| Nunavut | Nunavut Department of Health and Social Services | Yes | None | N/A |
|  | Public Health Department of the Cree Health Board | Yes | None | N/A |
